# Genetic Characterization and Evolutionary Insights of Novel H1N1 Swine Influenza Viruses Identified from Pigs in Shandong Province, China

**DOI:** 10.3390/v18010117

**Published:** 2026-01-15

**Authors:** Zhen Yuan, Ran Wei, Rui Shang, Huixia Zhang, Kaihui Cheng, Sisi Ma, Lei Zhou, Zhijun Yu

**Affiliations:** 1 School of Biological Science and Technology, University of Jinan, Jinan 250022, China; 17598037201@163.com (Z.Y.); bio_zhoul@ujn.edu.cn (L.Z.); 2 Poultry Institute, Shandong Academy of Agricultural Sciences, Jinan 250100, China; alexwran@163.com (R.W.); 1020226008@tju.edu.cn (H.Z.); 3 Shandong Provincial Key Laboratory of Livestock and Poultry Breeding (PKL2024B15), Jinan 250100, China; 4 College of Life Sciences, Shandong Normal University, Jinan 250014, China; 13573759287@163.com; 5 Institute of Animal Science and Veterinary Medicine, Shandong Academy of Agricultural Sciences, Jinan 250100, China; chengkaihui@126.com; 6 School of Animal Science and Technology, Ningxia University, Lanzhou 750021, China; 18561169291@163.com

**Keywords:** emerging infectious disease, H1N1, influenza viruses, swine, phylogenetic analysis

## Abstract

Influenza A viruses exhibit broad host tropism, infecting multiple species including humans, avian species, and swine. Swine influenza virus (SIV), while primarily circulating in porcine populations, demonstrates zoonotic potential with sporadic human infections. In this investigation, we identified two H1N1 subtype swine influenza A virus strains designated A/swine/China/SD6591/2019(H1N1) (abbreviated SD6591) and A/swine/China/SD6592/2019(H1N1) (abbreviated SD6592) in Shandong Province, China. The GenBank accession numbers of the SD6591 viral gene segments are PV464931-PV464938, and the GenBank accession numbers corresponding to each of the eight SD6592 viral gene segments are PV464939-PV464946. Phylogenetic and recombination analyses suggest potential evolutionary differences between the isolates. SD6591 displayed a unique triple-reassortant genotype: comparative nucleotide homology assessments demonstrated that the PB2, PB1, NP, NA, HA, and NEP genes shared the highest similarity with classical swine-origin H1N1 viruses. In contrast, SD6592 maintained genomic conservation with previously characterized H1N1 swine strains, although neither of these two isolates exhibited significant intrasegmental recombination events. Through comprehensive sequence analysis of these H1N1 SIVs, this study provides preliminary insights into their evolutionary history and underscores the persistent risk of cross-species transmission at the human–swine interface. These findings establish an essential foundation for enhancing national SIV surveillance programs and informing evidence-based prevention strategies against emerging influenza threats.

## 1. Introduction

Influenza A viruses are categorized by 18 hemagglutinin (H) subtypes and 11 neuraminidase (N) subtypes, forming 198 potential combinations. Among these, H1N1 represents one of the most prevalent subtypes [1]. Four key subtypes—H1N1, H2N2, H3N2, and H5N1—have demonstrated substantial impacts on human health. The 2009 influenza A (H1N1) pandemic originated from a novel H1N1 strain containing genetic material from swine, avian, and human influenza viruses. This triple-reassortant viral architecture enabled cross-species transmission, allowing simultaneous infection of humans, birds, and swine [2,3,4,5]. As a recombinant virus, it exhibits accelerated mutation rates and broad population susceptibility, driving recurrent global outbreaks that threaten public health security [6]. However, despite its recognized importance, systematic molecular surveillance data for swine influenza viruses in many regions, especially in pig-intensive areas, is still lacking. This surveillance gap limits our ability to understand the dynamics of virus evolution in real time, assess the risk of emerging novel recombinant strains, and provide early warnings of potential zoonotic threats. Therefore, conducting sustained and in-depth localized molecular epidemiological studies on viruses is a crucial step in translating the theoretical risks of the ‘mixing vessel’ concept into specific prevention and control insights. This study characterized two H1N1 influenza virus strains identified during routine epidemiological surveillance of swine herds in Shandong Province. Using high-throughput sequencing and phylogenetic analysis, we evaluated their genetic evolution to gain insights into their potential transmission pathways dynamics and inform targeted prevention strategies for mitigating public health and agricultural risks.

## 2. Materials and Methods

### 2.1. Sample Processing and Sequencing

In 2019, 200 porcine lung samples were obtained from swine herds exhibiting clinical signs of respiratory disease (coughing, fever, ocular/nasal discharge) across multiple cities in Shandong Province, China, including Liaocheng, Linyi, Jinan, Qingdao, and etc. All pig lung samples were independently collected by farmers in 2019 from deceased pigs exhibiting respiratory symptoms (fever, coughing, etc.) at different times and locations within their respective farms. These samples were subsequently sent to our laboratory for testing.

Lung samples were homogenized in 500 μL PBS buffer [7]. Following centrifugation at 10,000× *g* for 3 min to remove cellular debris, viral RNA was isolated from 200 μL of clarified supernatant using the Simply P Total RNA Extraction Kit (Bioflux, Hangzhou, China) per standardized protocols. All procedures were conducted within a biosafety cabinet using filter-tipped pipette tips to minimize aerosol contamination and cross-contamination.

This study employed validated universal primers from our laboratory for preliminary influenza virus detection. Following RT-PCR testing of 200 collected pig lung samples, two samples (1%) tested positive for SIV. These two positive samples originated from two pig farms in Linyi city and Shanghe County of Jinan City, Shandong Province. Positive samples were then sent to the company for next-generation sequencing to obtain full genome sequences.

### 2.2. Bioinformatic and Phylogenetic Analysis

The genomic characterization of H1N1 subtype swine influenza viruses was conducted through analysis of sequences obtained using MegAlign (version 7.1.0) and MEGA 11, where each gene fragment derived from sequencing served as the target sequence for subsequent homology-based investigations. High-homology sequences retrieved via BLAST (version 2.17.0) analysis were systematically compared with reference sequences acquired from NCBI GenBank (Table S1), encompassing human, canine, avian, and livestock origins, all specimens being temporally constrained to the 2004–2020 surveillance period.

Phylogenetic analysis of genes such as PB1 and PB2 was performed using MEGA11. The coding nucleotide sequences corresponding to the genes were downloaded from the NCBI database. Subsequent analyses were conducted using MEGA 11 software. First, the built-in MUSCLE algorithm was employed for multiple sequence alignment, followed by manual refinement to ensure correct codon framing. Subsequently, MEGA’s ‘Find Best Model’ function was employed to determine the GTR model as the optimal nucleotide substitution model for this dataset based on maximum likelihood principles. Finally, a phylogenetic tree was constructed using maximum likelihood analysis with the selected model, and bootstrap analysis with 1000 repetitions was performed to assess branch reliability.

### 2.3. Genetic Recombination Analysis

Potential recombination events were analyzed using SimPlot (version 3.5.1) and RDP5 (version 5.2) software and validated through 1000 bootstrap repetitions. In the RDP5 analysis, recombination signals were determined using a corrected *p*-value threshold. The selection of reference strains is based on the following principles: Sequence similarity and priority is given to strains exhibiting the highest homology with each gene fragment of the strain under study in BLAST alignments.

### 2.4. In Vitro Replication Experiment of Homologous Virus Isolates

To verify the potential biological functions of the virus sequences tested in this study, we used an H1N1 isolate (A/swine/China/Qingdao/2018, abbreviated as 2018-H1N1, GenBank accession number: MK587711-MK587718) that is highly homologous to the SD6591 and SD6592 strains in this study for in vitro replication ability evaluation [8], and used PR8 virus (A/Puerto Rico/8/34 (H1N1)) as a control. Three cell lines, PK-15 (pig kidney epithelium), MDCK (dog kidney epithelium), and A549 (human lung glandular epithelium), were selected for the experiment. DMEM (PK-15, MDCK) or F12K (A549) medium containing 10% fetal bovine serum and 1% bispecific antibody were used for cultivation at 37 °C and 5% CO_2_. When the cells grow to a confluence degree of over 90%, they are inoculated with 2018-H1N1 or PR8 viruses at a multiplicity of infection (MOI) of 0.1. After adsorption for 1 h, they are replaced with the corresponding maintenance medium containing 2% TPCK trypsin. The experiment was divided into two parts: protein level and RNA level. Cell pellet was collected 12 h after infection for Western blot detection of viral protein expression; Collect cell pellet at 12 and 24 h after infection for qRT PCR analysis of viral RNA replication kinetics. In Western blot detection, an equal amount of total protein was taken after cell lysis for SDS-PAGE and membrane transfer. Rabbit anti influenza virus NP (GTX636199, GeneTex, 1:2000 dilution) and PB1 (GTX125923, GeneTex, 1:2000 dilution) monoclonal antibodies were used as primary antibodies, HRP labeled goat anti rabbit IgG was used as secondary antibodies (F300808, Abways, 1:5000 dilution), and GAPDH (AB0036, Abways, 1:1000 dilution) was used as an internal reference to compare the protein expression differences between the infected group and the control group. In qRT PCR analysis, total RNA was extracted using the Accurate Biology Rapid RNA Extraction Kit. Use the company’s one-step RT qPCR kit and influenza virus conserved gene specific primer probes for amplification detection (specific primers are shown in Table 1), and use in vitro transcribed RNA as a standard for absolute quantification to calculate the virus RNA copy number at each time point. Repeat all experiments using three techniques. Use GraphPad Prism software (version 9.0.0) for data statistical analysis. Using *t*-test for inter group comparison, * *p* < 0.05 indicates statistically significant differences.

## 3. Results

### 3.1. Virus Gene Sequencing and Sequence Submission

This study collected 200 swine lung tissue samples from multiple locations in Shandong Province. Two distinct influenza A viruses were identified and sequenced from these samples, designated as A/swine/China/SD6591/2019(H1N1) (abbreviated as SD6591) and A/swine/China/SD6592/2019(H1N1) (abbreviated as SD6592).

The GenBank accession numbers of the SD6591 viral gene segments are PV464931-PV464938, and the GenBank accession numbers corresponding to each of the eight SD6592 viral gene segments are PV464939-PV464946.

### 3.2. Bioinformatic Analysis

We analyzed the individual viral genes using the BLAST database, followed by comparative assessment of nucleotide homology between SD6591 and other H1N1 strains available in GenBank. The PB2, PB1, NP, NA, HA, and NEP genes exhibited the highest homology with swine-origin H1N1 subtype influenza viruses. The PA and M2 genes exhibit high homology with human-origin H1N1 subtype influenza viruses, indicating that the PA and M2 genes of this virus are most closely related to the corresponding gene clusters of human seasonal H1N1 influenza viruses. This suggests that these gene fragments may ultimately originate from human H1N1 viruses. However, no direct epidemiological evidence currently links this virus to specific recent human cases. This genetic association suggests ongoing evolution of human-derived viral genes within swine populations and underscores the importance of monitoring swine influenza for zoonotic transmission risks (Table 2). Isolates from humans and dogs are tightly nested within the evolutionary branch of swine viruses, lacking evidence of independent multigenerational transmission. This aligns with the genetic pattern of unidirectional spillover from pigs to humans and from pigs to dogs. Therefore, it is considered a zoonotic event, representing an instance where a virus circulating within a pig population was captured upon spilling over to an accidental host.

This genomic analysis indicates that strain SD6591 potentially represents a human–swine influenza reassortant virus. Phylogenetic analysis indicates that the M2 gene of strain SD6591 is most closely related to canine-origin H1N1 influenza viruses, suggesting this gene fragment may have entered swine populations through cross-species transmission between dogs and pigs. Selecting human-origin strains as references aims to establish a standard host reference system and conduct preliminary public health risk assessments. This finding reveals the complexity of influenza virus transmission pathways across species and underscores the necessity of expanding surveillance to include companion animals.

Subsequent comparative genomic characterization of SD6592 through GenBank alignment revealed that PB2, PB1, NP, NA, HA, NEP, PA, and M2 gene segments exhibited >98% nucleotide homology with H1N1 subtype swine influenza strains, suggesting limited genetic divergence between the newly identified strain and previously documented H1N1 variants. Notably, phylogenetic analysis demonstrated closest evolutionary relationship with A/swine/Beijing/0301/2018(H1N1) (Table 3).

### 3.3. Phylogenetic Analysis

**Figure 1:**
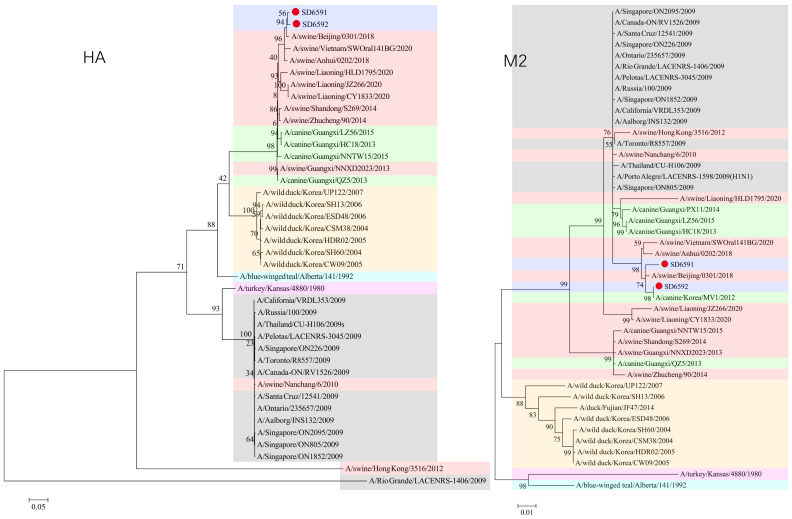
Phylogenetic tree based on HA, M2 gene of the two viruses.

**Figure 2:**
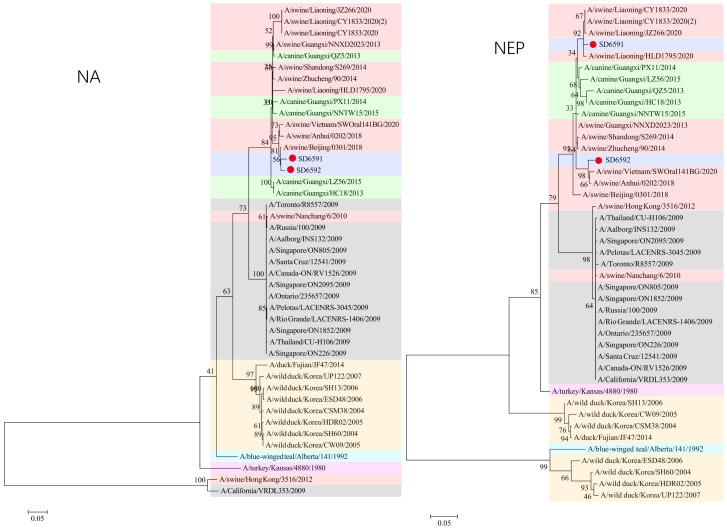
Phylogenetic tree based on NA, NEP gene of the two viruses.

**Figure 3:**
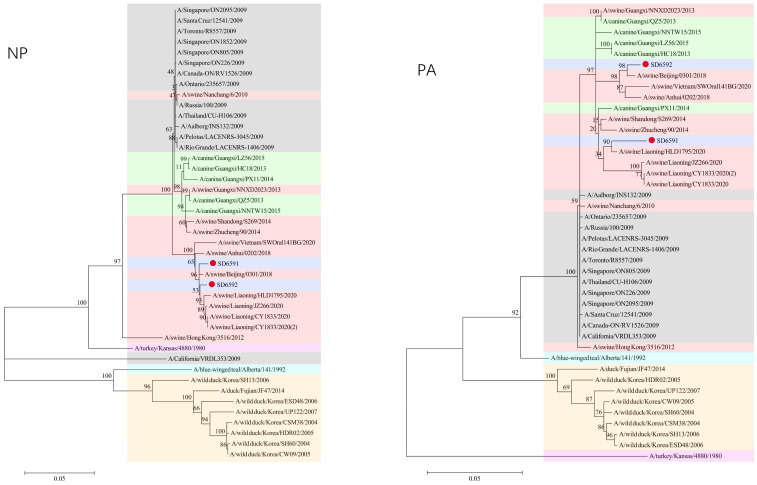
Phylogenetic tree based on NP, PAgene of the two viruses.

**Figure 4:**
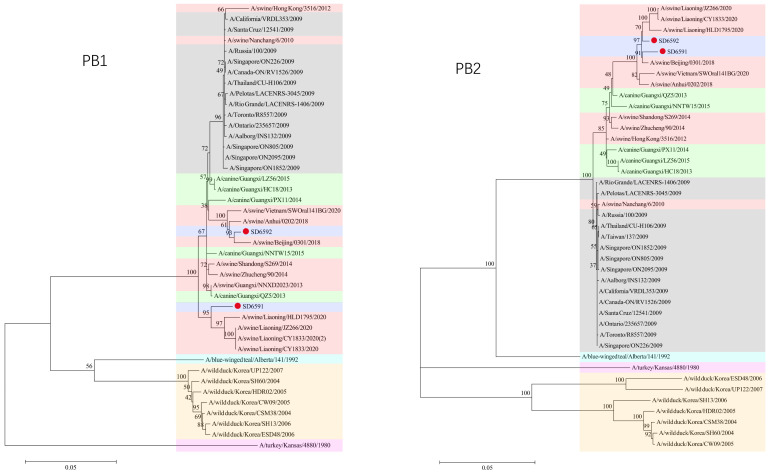
Phylogenetic tree based on PB1, PB2 gene of the two viruses.

We conducted phylogenetic analysis of influenza virus strains SD6591 and SD6592 through individual gene alignment. As shown in Figure 1, Figure 2, Figure 3 and Figure 4, the HA, NA, NP, and PB2 genes of SD6591 clustered with A/swine/Beijing/0301/2018(H1N1), while PA and NEP genes shared higher similarity with the same reference strain. The M2 gene displayed dual phylogenetic affinity with both A/swine/Beijing/0301/2018(H1N1) and A/canine/Korea/MV1/2012. The PB1 gene demonstrated evolutionary proximity to A/swine/Liaoning/CY1833/2020, A/swine/Liaoning/HLD1795/2020, and A/swine/Liaoning/JZ266/2020.

As shown in Figure 1, Figure 2, Figure 3 and Figure 4, analysis shows that the HA, PA, NA, and PB1 genes of SD6592 form a monophyletic branch with A/swine/Beijing/0301/2018(H1N1), while M2 is more closely related to A/canine/Korea/MV1/2012. The NEP gene shares ancestral connections with A/swine/Vietnam/SWOral141BG/2020 and A/swine/Anhui/0202/2018, while PB2 and NP genes cluster with Liaoning swine variants CY1833/2020, HLD1795/2020, and JZ266/2020. The NA gene forms a polyphyletic group encompassing H1N1 swine influenza variants from Vietnam, Anhui, and Beijing. Red solid dots: Represent original viral sequences sequenced or analyzed in this study. Branch node numbers: Indicate bootstrap support values, used to assess the confidence and reliability of each branch (calculated through 1000 repeated bootstrap samples).

### 3.4. Genetic Recombination Analysis

**Figure 5:**
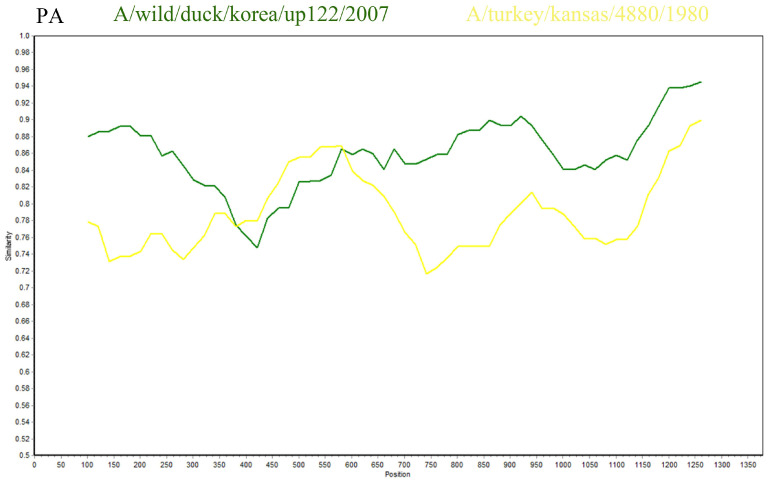
Results of recombination analysis of SD6591 PA gene. (The horizontal axis represents nucleotide position, and the vertical axis represents similarity).

H1N1 has been detected in a variety of mammals and it has the potential for zoonotic transmission [9]. In addition, genetic recombination is an important mode of RNA virus evolution [10]. This study utilized SimPlot software (verison 3.5.1), with SD6591 as the query sequence, to conduct recombination analysis with the A/Wild duck/Korea/UP122/2007 strain and the A/turkey/Kansas/4880/1980 strain. The results indicated that the PA gene of SD6591 exhibited a high degree of sequence homology (87.5%) with the A/turkey/Kansas/4880/1980 strain (GenBank accession number: EU742643.2) within the 400–600 bp region, while it showed even higher homology (95.2%) with the A/Wild duck/Korea/UP122/2007 strain (GenBank accession number: HQ014821.1) within the 600–1250 bp region. This suggests that the PA gene of SD6591 may originate from a recombination event between the aforementioned duck-derived and turkey-derived strains (Figure 5).

**Figure 6:**
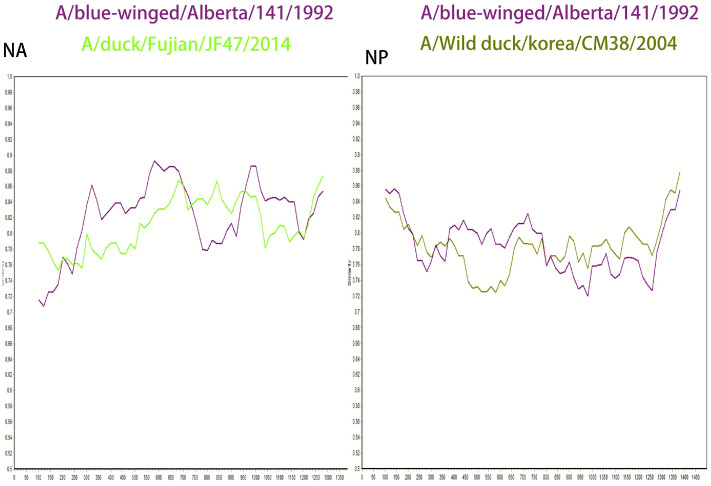
Results of recombination analysis of SD6592 NA and NP genes. (The horizontal axis represents nucleotide position, and the vertical axis represents similarity).

In addition, this study conducted a SimPlot recombination analysis of the NA gene using SD6592 as the query sequence, with the A/blue-winged teal/Alberta/141/199 strain and the A/duck/Fujian/JF47/2014 strain as backgrounds. The results showed that the sequence homology was relatively high (89.2%) in the 300–750 bp interval with the A/blue-winged teal/Alberta/141/199 strain, while the homology was higher (85.5%) in the 650–900 bp interval with the A/duck/Fujian/JF47/2014 strain (GenBank accession number: KP657996.1), suggesting that the NA gene may originate from recombination between the two aforementioned strains (Figure 6).

In the NP gene analysis, recombination analysis was conducted using the A/blue-winged teal/Alberta/141/199 strain (GenBank accession number: CY004545.1) and the A/wild duck/Korea/CSM38/2004 strain (GenBank accession number: HQ014821.1) as backgrounds. The results indicated a high homology (82.5%) with the A/blue-winged teal/Alberta/141/199 strain in the 400–700 bp region, and a high homology (82.5%) with the A/wild duck/Korea/CSM38/2004 strain in the 850–1300 bp region, suggesting that the NP gene may have been formed by recombination of the aforementioned two wild duck-derived strains (Figure 6).

To confirm the evolutionary history of the PA, NA and NP gene, we conducted a multi-algorithm recombination event analysis using the RDP5 software package, building upon our preliminary SimPlot analysis. After testing with multiple methods including RDP, GENECONV, and BootScan, and applying a corrected *p*-value threshold of <0.05, no statistically significant recombination signals were detected. These results indicate that the sequence mosaicism observed in the PA gene is more likely attributable to complex point mutation accumulation or unsampled ancestral sequences, rather than recent direct recombination events. This finding suggests relative genetic stability within this viral lineage in the porcine host.

### 3.5. High Homology and In Vitro Replication Ability of Homologous Isolates

**Figure 7:**
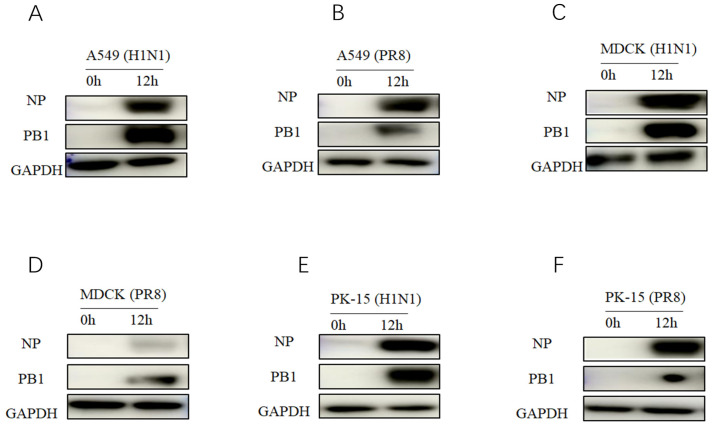
Western blot analysis of PB1 and NP protein expression in three cell lines infected with 2018-H1N1 or PR8 virus. Cells were infected at an MOI of 0.1, and whole-cell lysates were collected at 0 and 12 h post-infection (hpi). Protein levels were detected using antibodies against PB1, NP, and GAPDH (loading control). (**A**,**B**) A549 cells. (**C**,**D**) MDCK cells. (**E**,**F**) PK-15 cells.

**Figure 8:**
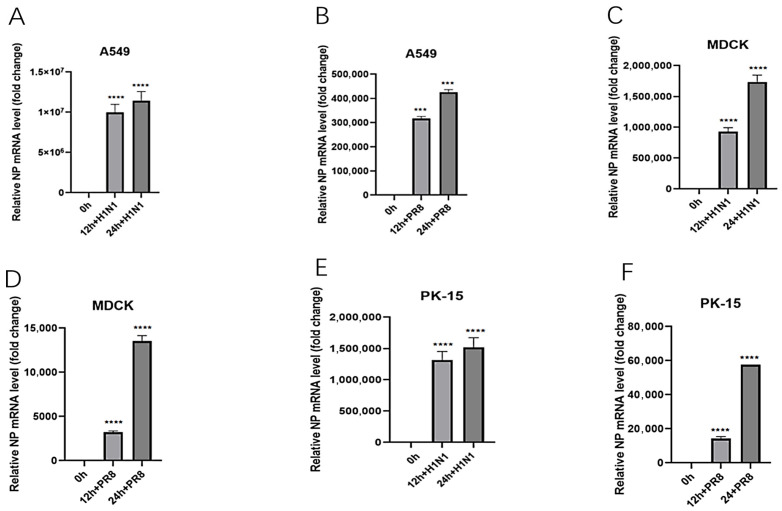
Replication kinetics of 2018-H1N1 and PR8 viruses in different cell lines. Cells were infected at an MOI of 0.1, and viral RNA was quantified by absolute qRT-PCR targeting the NP gene at the indicated time points. Data represent mean ± SD from three independent experiments. (**A**,**B**) A549 cells. (**C**,**D**) MDCK cells. (**E**,**F**) PK-15 cells. Statistical significance was analyzed using Student’s t-test. *** indicates *p* < 0.0003, and **** indicates *p* < 0.0001.

The two strains sequenced in this study were subjected to homology analysis with the 2018-H1N1 and PR8 influenza viruses. Among the gene segments, the highly conserved PA and M2 genes had the highest homology (>96%), while the homology of the NA gene was slightly lower but still higher than 91.0% (Table 4 and Table 5). This result confirms genetically that the virus identified in this study is highly homologous to the active 2018-H1N1 isolate. Based on this high degree of homology, we used the 2018-H1N1 isolate as a substitute virus to evaluate the replication potential of the virus identified in this study. This virus exhibits efficient replication ability in PK-15, MDCK, and A549 cells. Protein immunoblotting analysis confirmed that there was abundant expression of virus PB1 and NP proteins in infected cells 12 h after infection (Figure 7). Consistent with protein level data, qRT PCR analysis showed that the copy number of viral RNA increased over time in the sediment of all cell lines, reaching its peak at 24 h after infection (Figure 8). This replication trend is similar to the control PR8 virus. These results collectively indicate that viruses with high genetic similarity to the samples tested in this study are capable of effective in vitro replication in various mammalian cell types.

## 4. Discussion

SIV can cause highly contagious respiratory diseases in pigs, it has led to an increase in mortality rates on many farms in China, posing a major threat to the farming industry in China [11,12]. H1N1 subtypes of swine influenza viruses are widespread in swine populations and undergo regular mutation. In 2009, a novel H1N1 swine influenza virus was first detected in Mexico and rapidly spread worldwide, triggering the so-called “H1N1 influenza A pandemic” [13]. This pandemic demonstrated that swine influenza viruses can spread across species to humans and cause global epidemics. Currently, the spread of influenza can be prevented and controlled through enhanced vaccination, personal hygiene and public health measures [14].

This study detected only 2 SIV-positive cases (a positivity rate of 1%) among 200 samples. However, this investigation involved nucleic acid testing only. We will subsequently collect serum samples and conduct hemagglutination inhibition tests to verify whether the virus is prevalent in pig populations. Pigs act as a “mixing vessel” for influenza viruses, continuous monitoring and research on swine influenza viruses is also an important means to detect new mutated strains in time and develop effective preventive and control measures. Analysis of the biological characteristics of two H1N1 subtypes, SIV SD6591 and SD6592, identified in two independent infected pigs in Shandong Province, aims to understand the epidemiological situation of SIV in Shandong pig farms and provide reference for the prevention and control of swine influenza in China.

In the present study of viruses from Shandong, homology analysis showed that SD6591 had high nucleotide sequence homology with SIV identified in Beijing or Anhui, and the nucleotide sequences with the highest homology in the PA and M2 genes were originated from human beings, To validate this reassortment hypothesis and elucidate its biological significance, future research urgently requires: conducting mixed infection studies under experimental conditions to assess the likelihood and efficiency of reassortment occurring when human influenza viruses and swine influenza viruses co-infect cells or animal models.

As an important component of viral RNA polymerase, the sequence variation in PA protein directly affects replication efficiency and host adaptability. Studies have shown that the PA gene of human-derived influenza viruses often contains specific adaptive mutations [15]. The human-derived PA fragment obtained in SD6591 may endow the virus with more efficient replication potential in pigs and provide a molecular basis for potential cross-species transmission between humans and pigs. Secondly, M2 ion channel protein plays a central role in the virus uncoating process and is also the target of the classic antiviral drug amantadine. Homology analysis and phylogenetic analysis of the M2 gene of the SD6591 strain in this study reveal that it clusters with recent human and canine influenza virus lineages on the same evolutionary branch. This finding is particularly noteworthy because since the 2000s, viruses from these lineages (including the globally circulating seasonal H1N1 and the 2009 pandemic H1N1 viruses) have generally developed extensive resistance to amantadine-based drugs due to mutations such as S31N in the M2 protein [16]. Therefore, the specific genetic background embedded in SD6591 itself serves as an important risk signal, clearly indicating the priority directions for subsequent functional verification. Although the main focus of this study is on comprehensive genomic characterization and evolutionary tracing, the aforementioned findings have provided key genotypic clues for in-depth evaluation of its potential drug sensitivity. Future monitoring and research can build on this foundation by conducting targeted sequencing and functional characterization of the transmembrane region of the M2 protein to determine whether similar resistance characteristics exist. If confirmed, such strains may have pre-existing resistance to some traditional antiviral treatment regimens, highlighting the necessity for continuous monitoring and the clinical reserve of a new generation of antiviral drugs.

Corresponding to the “novelty” of SD6591, the “conservativeness” represented by SD6592 also carries significant epidemiological implications. Its high genomic conservativeness indicates that a certain classical swine influenza virus lineage has established a stable transmission chain within local pig populations and occupied a sustained ecological niche. Such a stable circulating strain typically exhibits optimized host adaptability, effectively spreading within pig populations and forming a concealed and stable “genetic reservoir”. The risk posed by this “genetic reservoir” is twofold: firstly, it itself continues to cause animal health and economic losses as a pathogen; secondly, and more critically, it provides an excellent “reassortment partner” for novel reassortant viruses like SD6591. When a novel invading strain (such as SD6591) co-infects the same host with such long-circulating local strains (such as SD6592), the probability of genetic reassortment between them greatly increases, potentially giving rise to offspring that combine “novelty” and “adaptability”, thereby significantly elevating the risk of generating pandemic-potential strains.

The establishment of a biological phylogenetic tree for each gene by MEGA11 showed that strain SD6591 was closely related to A/swine/Beijing/0301/2018 (H1N1) and A/canine/Korea/MV1/-2012 (H1N1), thus verifying the cross-species transmission of SIV [17]. When two or three viral strains infect the same host at the same time, their genome segments may be exchanged and recombined, and genetic recombination can produce influenza virus strains with new characteristics. Swine is one of the most important hosts for the genetic recombination of influenza viruses, and these strains of viruses may have a higher pathogenicity or a stronger transmission capacity [18,19,20]. Therefore, in-depth studies of genetic mutations and genetic recombination of SIV are important for understanding their pathogenic mechanisms, developing effective prevention and control strategies, and developing novel vaccines and antiviral drugs [21].

Although preliminary analysis based on similarity graphs suggested sequence differences, more rigorous RDP5 multi-algorithm reassortment analysis failed to reveal clear statistical evidence supporting reassortment events. Therefore, the observed sequence variations are more likely attributable to accumulated point mutations rather than reassortment. This finding suggests that this viral lineage may exhibit relative genetic stability during evolution, which is significant for understanding its epidemiological characteristics.

The observations in this study are based on a limited sample size; therefore, the evolutionary differences revealed are preliminary and require confirmation through future, more extensive research. Furthermore, this study is based on genomic analysis and has not yet involved experimental verification of viral biological characteristics (such as replication capacity and pathogenicity) or real-world epidemiological conditions. Future comprehensive assessments of its potential risks should be conducted through viral isolation, animal experiments, and large-scale epidemiological investigations.

The experimental design mainly focused on virus genome sequencing of the samples, but failed to attempt to isolate live viruses from these samples at the same time. The attempts to supplement and separate the samples in the later stage were also unsuccessful due to prolonged sample storage time and possible loss of virus vitality. Therefore, the initial core findings of the study were limited to features at the gene sequence level.

However, to overcome this limitation and infer the functional characteristics of the virus we sequenced, we utilized a highly homologous and active isolate preserved in the laboratory. The extremely high genetic homology indicates that a swine influenza virus named A/swine/China/Qingdao/2018 (H1N1) isolated from swine in 2018 is a functional representative of the virus detected in this study. Subsequent in vitro experiments have shown that this represents the effective replication of the virus in pig, dog, and human derived cells. This discovery is of great significance, as the replication ability of the virus in A549 cells (human lung epithelial cell model) highlights its potential to infect human cells, and further research is necessary to investigate its cross-species transmission risk. Future research will prioritize isolating contemporaneous prevalent strains from fresh samples for direct phenotypic analysis, including pathogenicity and transmissibility assessment.

## 5. Conclusions

This study conducted a systematic genetic analysis of eight gene fragments of two strains of swine influenza virus, SD6591 and SD6592, including homology comparison, phylogenetic tree construction, and recombination detection. The sequence analysis results showed that no significant recombination events were observed in the gene fragments, indicating that these two viruses maintained a relatively stable genetic background during this study.

More importantly, in order to go beyond the limitations of sequence analysis and directly evaluate the potential biological characteristics of these viruses, we introduced isolated strains that are highly homologous (>91%) to our study strain for in vitro functional validation. Cell infection experiments have confirmed that the homologous virus has efficient replication ability in porcine (PK-15), canine (MDCK), and human (A549) epithelial cells. This provides direct experimental evidence for its potential host adaptability and public health risks.

In summary, this study not only completed the genetic analysis of two strains of swine influenza virus, but also successfully associated them with in vitro replication phenotype through an innovative “homologous virus functional substitution” strategy. Ultimately, this study emphasizes the core role of pigs as “mixing containers” for influenza viruses: even in the “stable” stage where significant genetic recombination is not detected, circulating influenza viruses in pigs may still retain their inherent potential to infect multiple host cells. Therefore, the dual track strategy of continuously strengthening the “genome monitoring” of influenza viruses in pig herds and “in vitro phenotype verification of representative strains” is of great scientific and practical significance for timely detection of potential adaptive mutations or strains with new characteristics, and for early warning and prevention of new influenza epidemics.

## Figures and Tables

**Table 1:** Primer sequences used for quantitative real-time PCR.

Gene Name	Primer Sequence (5’→ 3’)
A549-GAPDH-Q-F	ACCCACTCCTCCACCTTTGA
A549-GAPDH-Q-R	TGTTGCTGTAGCCAAATTCGTT
MDCK-GAPDH-Q-F	GAAGGTCGGAGTGAACGGATTT
MDCK-GAPDH-Q-R	TGGGTGGAATCATACTGGAACA
PK-15-GAPDH-Q-F	GAGTCAACGGATTTGGTCGT
PK-15-GAPDH-Q-R	TTGATTTTGGAGGGATCTCG
NP(H1N1)-Q-F	GGGTGAAAATGGTCGAAGGACA
NP(H1N1)-Q-R	ATACACACAAGCAGGCAGGCAG

**Table 2:** Strains with the closest homology to each gene of SD6591.

Gene	Virus with the Highest Nucleotide Identity	Accession No.	Identity
PB2	A/swine/Beijing/0301/2018(H1N1)	MN416329.1	98.88%
PB1	A/swine/Anhui/HD21/2020(H1N1)	OL744679.1	97.65%
NA	A/swine/Beijing/0301/2018(H1N1)	MN416693.1	98.30%
PA	A/Hong Kong/H090-721-V10/2009(H1N1)	CY111816.1	96.99%
NP	A/swine/Beijing/0301/2018(H1N1)	MN418733.1	99.13%
HA M2 NEP	A/swine/China/S69-kid/2018(H1N1)	OM149829.1	98.98%
	A/Porto Alegre/LACENRS-1598/2009(H1N1)	KY925142.1	97.93%
	A/swine/Liaoning/CY1833/2020(H1N1)	OL311131.1	98.96%
	A/swine/Beijing/0301/2018(H1N1)	MN416329.1	98.88%

**Table 3:** Strains with closest homology to SD6592.

Gene	Virus with the Highest Nucleotide Identity	Accession No.	Identity
PB2	A/swine/Beijing/0301/2018(H1N1)	MN416329.1	99.25%
PB1	A/swine/Beijing/0301/2018(H1N1)	MN416416.1	98.94%
NP	A/swine/Beijing/0301/2018(H1N1)	MN418733.1	99.20%
NA	A/swine/Beijing/0301/2018(H1N1)	MN416693.1	98.68%
PA	A/swine/Beijing/0301/2018(H1N1)	MN416516.1	98.70%
HA	A/swine/Shandong/LY059/2019(H1N1)	MW127000.1	98.53%
M2	A/swine/Shandong/LY059/2019(H1N1)	MW127003.1	99.29%
NEP	A/swine/Anhui/0202/2018(H1N1)	MN418656.1	98.81%

**Table 4:** Nucleotide identity (%) of SD6591 gene segments with two reference H1N1 strains.

Gene	Identity	Accession No.	Identity
PB2	A/swine/China/Qingdao/2018(H1N1)	MK587711	94.37%
	A/Puerto Rico/8/1934(H1N1)	NC_002023.1	84.99%
PB1	A/swine/China/Qingdao/2018(H1N1)	MK587712	93.86%
	A/Puerto Rico/8/1934(H1N1)	NC_002021.1	81.75%
NA	A/swine/China/Qingdao/2018(H1N1)	MK587716	91.06%
	A/Puerto Rico/8/1934(H1N1)	NC_002018.1	81.43%
PA	A/swine/China/Qingdao/2018(H1N1)	MK587713	97.79%
	A/Puerto Rico/8/1934(H1N1)	NC_002022.1	82.38%
NP	A/swine/China/Qingdao/2018(H1N1)	MK587715	94.86%
	A/Puerto Rico/8/1934(H1N1)	NC_002019.1	85.48%
HA	A/swine/China/Qingdao/2018(H1N1)	MK587714	92.76%
	A/Puerto Rico/8/1934(H1N1)	NC_002017.1	75.84%
M2	A/swine/China/Qingdao/2018(H1N1)	MK587717	96.54%
	A/Puerto Rico/8/1934(H1N1)	NC_002016.1	89.31%
NEP	A/swine/China/Qingdao/2018(H1N1)	MK587718	97.93%
	A/Puerto Rico/8/1934(H1N1)	NC_002020.1	82.73%

**Table 5:** Nucleotide identity (%) of SD6592 gene segments with two reference H1N1 strains.

Gene	Identity	Accession No.	Identity
PB2	A/swine/China/Qingdao/2018(H1N1)	MK587711	94.91%
	A/Puerto Rico/8/1934(H1N1)	NC_002023.1	85.19%
PB1	A/swine/China/Qingdao/2018(H1N1)	MK587712	94.71%
	A/Puerto Rico/8/1934(H1N1)	NC_002021.1	82.45%
NA	A/swine/China/Qingdao/2018(H1N1)	MK587716	91.43%
	A/Puerto Rico/8/1934(H1N1)	NC_002018.1	81.94%
PA	A/swine/China/Qingdao/2018(H1N1)	MK587713	96.75%
	A/Puerto Rico/8/1934(H1N1)	NC_002022.1	83.18%
NP	A/swine/China/Qingdao/2018(H1N1)	MK587715	94.92%
	A/Puerto Rico/8/1934(H1N1)	NC_002019.1	85.69%
HA	A/swine/China/Qingdao/2018(H1N1)	MK587714	92.95%
	A/Puerto Rico/8/1934(H1N1)	NC_002017.1	74.97%
M2	A/swine/China/Qingdao/2018(H1N1)	MK587717	96.64%
	A/Puerto Rico/8/1934(H1N1)	NC_002016.1	89.31%
NEP	A/swine/China/Qingdao/2018(H1N1)	MK587718	95.23%
	A/Puerto Rico/8/1934(H1N1)	NC_002020.1	83.81%

## Data Availability

The original contributions presented in this study are included in the article/Supplementary Material. Further inquiries can be directed to the corresponding author.

## Supplementary Materials

The following supporting information can be downloaded at: https://www.mdpi.com/article/10.3390/v18010117/s1, Table S1: Sequences of the constructed genes (PB2 as an example).
