# Supplementary material for: Genetic Characterization and Evolutionary Insights of Novel H1N1 Swine Influenza Viruses Identified from Pigs in Shandong Province, China
Source: Viruses. 2026 Jan 15;18(1):117. doi: 10.3390/v18010117 (PMC12846427; doi:10.3390/v18010117)
Supplement: Supplementary file 1 [file viruses-18-00117-s001.zip › viruses-4064909-supplementary.pdf]

The novel recombinant swine influenza viruses infection in pigs in Shandong Province, China

Technical Appendix

Supplementary Table 1 Sequences of the constructed genes (PB2 as an example)

Gene	Virus with the highest nucleotide identity	Accession no.
PB2	A/swine/Beijing/0301/2018	MN416329.1
PB2	A/swine/Vietnam/SWOral141BG/2020	PP751647.1
PB2	A/canine/Guangxi/QZ5/2013	MG254172.1
PB2	A/swine/Hong Kong/3516/2012	ON850188.1
PB2	A/swine/Shandong/S269/2014	KP735703.1
PB2	A/canine/Guangxi/PX11/2014	MG254185.1
PB2	A/Singapore/ON805/2009	CY124076.1
PB2	A/Singapore/ON1852/2009	CY123553.1
PB2	A/swine/Nanchang/6/2010	JF275948.1
PB2	A/Russia/100/2009	CY054659.1
PB2	A/Toronto/R8557/2009	GQ502903.1
PB2	A/swine/Anhui/0202/2018	MN416326.1
PB2	A/Pelotas/LACENRS-3045/2009	KY925018.1
PB2	A/swine/Liaoning/JZ266/2020	OL310979.1
PB2	A/Thailand/CU-H106/2009	GQ866916.2
PB2	A/Ontario/235657/2009	CY060523.1
PB2	A/swine/Liaoning/CY1833/2020	OL310971.1

PB2	A/swine/Liaoning/HLD1795/2020	OL310980.1
PB2	A/canine/Guangxi/NNTW15/2015	MG254184.1
PB2	A/canine/Guangxi/LZ56/2015	MG254182.1
PB2	A/Singapore/ON226/2009	CY123822.1
PB2	A/Taiwan/137/2009	CY045239.1
PB2	A/swine/Zhucheng/90/2014	KX264368.1
PB2	A/Singapore/ON2095/2009	CY123750.1
PB2	A/Canada-ON/RV1526/2009	GQ132137.1
PB2	A/California/VRDL353/2009	CY092782.1
PB2	A/canine/Guangxi/HC18/2013	MG254171.1
PB2	A/Aalborg/INS132/2009	CY083917.1
PB2	A/turkey/Kansas/4880/1980	EU742643.2
PB2	A/duck/Fujian/JF47/2014	KP657996.1
PB2	A/wild duck/Korea/CSM38/2004	HQ014741.1
PB2	A/wild duck/Korea/SH60/2004	HQ014757.1
PB2	A/wild duck/Korea/HDR02/2005	HQ014765.1
PB2	A/wild duck/Korea/CW09/2005	HQ014773.1
PB2	A/wild duck/Korea/SH13/2006	HQ014781.1
PB2	A/wild duck/Korea/ESD48/2006	HQ014813.1
PB2	A/wild duck/Korea/UP122/2007	HQ014821.1
PB2	A/blue-winged teal/Alberta/141/1992	CY004545.1
